# Endovascular aortic arch repair with chimney technique for pseudoaneurysm

**DOI:** 10.1186/s12872-023-03091-4

**Published:** 2023-02-12

**Authors:** Ming-yao Luo, Xiong Zhang, Kun Fang, Yuan-yuan Guo, Dong Chen, Jason T. Lee, Chang Shu

**Affiliations:** 1 State Key Laboratory of Cardiovascular Disease, Center of Vascular Surgery, Fuwai Hospital, National Center for Cardiovascular Diseases, Chinese Academy of Medical Sciences and Peking Union Medical College, No.167, Beilishi Road, Xicheng District, Beijing, 100037 China; 2 Department of Vascular Surgery, Fuwai Yunnan Cardiovascular Hospital, Affiliated Cardiovascular Hospital of Kunming Medical University, Kunming, 650102 China; 3 Department of Surgery, Stanford University School of Medicine, Stanford, CA 94305 USA; 4 Department of Vascular Surgery, The Second Xiangya Hospital of Central South University, Changsha, 410013 China

**Keywords:** Aortic arch pseudoaneurysm, Chimney graft/technique, Thoracic endovascular aortic repair, Type Ia endoleak, Trauma, Innominate artery, Left common carotid artery, Left subclavian artery

## Abstract

**Background:**

Aortic pseudoaneurysm is a life-threatening clinical condition, and thoracic endovascular aortic repair (TEVAR) has been reported to have a relatively satisfactory effect in aortic pathologies. We summarized our single-centre experience using chimney TEVAR for aortic arch pseudoaneurysms with inadequate landing zones.

**Methods:**

A retrospective study was conducted from October 2015 to August 2020, 32 patients with aortic arch pseudoaneurysms underwent chimney TEVAR to exclude an aortic lesion and reconstruct the supra-aortic branches, including 3 innominate artery, 12 left common carotid arteries and 29 left subclavian arteries. Follow-up computed tomography was suggested before discharge; at 3, 6, 12 months and yearly thereafter.

**Results:**

The median age of 32 patients was 68.0 years (range, 28–81) with the mean max diameter of aneurysm of 47.9 ± 12.0 mm. Forty-four related supra-aortic branches were well preserved, and the technical success rate was 100%. The Type Ia endoleaks occurred in 3 (9%) patients. Two patients were lost to follow-up and 4 patients died during the follow-up period. The mean follow-up times was 46.5 ± 14.3 months. One patient died due to acute myocardial infarction just 10 days after chimney TEVAR and the other 3 patients passed away at 1.5 months, 20 months, and 31 months with non-aortic reasons. The 4.5-year survival estimate was 84.4%. The primary patency rate of the target supra-arch branch vessels was 97.7% (43/44), and no other aorta-related reinterventions and severe complications occurred.

**Conclusion:**

For aortic arch pseudoaneurysms with inadequate landing zones for TEVAR, the chimney technique seems to be feasible, with acceptable mid-term outcomes, and it could serve as an alternative minimally invasive approach to extend the landing zone. Slow flow type Ia endoleak could be treated conservatively after chimney TEVAR. Additional experience is needed, and the long-term durability of chimney TEVAR requires further follow-up.

## Background

Aortic pseudoaneurysm is a life-threatening clinical condition, and the causes include penetrating aortic ulcer (PAU), trauma, iatrogenic aetiologies, Bechet’s disease, and aortic infections (mycotic aneurysms) [1]. The development of endovascular treatment in recent decades has provided a new treatment option for open surgery [2, 3]. Currently, as chimney or fenestration techniques are used as assistive techniques, the indications for TEVAR have notably expanded [4].

Since 2002, chimney TEVAR has been reported to be used successfully for different types of aortic arch diseases [5, 6]. However, for the treatment of aortic arch pseudoaneurysms, the current literature on chimney TEVAR is limited [7–18]. The aim of this retrospective study was to report the mid-term results of chimney TEVAR for aortic arch pseudoaneurysms in our centre.

## Methods

### Patients

From October 2015 to August 2020, 32 patients with aortic arch pseudoaneurysms underwent chimney TEVAR. All patients in this group received preoperative computed tomography angiography (CTA) of the aorta for diagnosis and measurement. The effective diameter, the average of the aortic anteroposterior and lateral diameters, was independently measured by two radiologists using 1-mm–collimation double-oblique reconstructions. The image sizing was conducted with Syngo fastView software (version VX57133, Siemens Healthineers, Germany). The decision regarding whether chimney TEVAR could be used was made based on the anatomic features of the pseudoaneurysm and the arch.

The present study was approved by the Ethics Committee of Fuwai Hospital (Approval No. 2021-1525), and the procedures were in accordance with the Declaration of Helsinki. Informed consent was waived because this was a retrospective study. Both open and endovascular procedures are routinely performed in our centre, and the choice of surgical approach generally depends on the assessment of the patient's comorbidities and wishes.

The inclusion criteria for the chimney technique for LSA were as follows: pseudoaneurysms close to (< 15 mm) or already involving the orifice of the left subclavian artery (LSA) and a distance longer than 15 mm between the orifice of the LSA and the left common carotid artery (LCCA). The inclusion criteria of the chimney technique for LCCA with or without LSA were as follows: pseudoaneurysms involved zone 1 and a distance longer than 15 mm between the orifice of the LCCA and the innominate artery (IA). The inclusion criteria of the chimney technique in the IA and LCCA, with or without LSA, were as follows: pseudoaneurysms involved zone o; patients had high risks for open surgery or refused open surgery.

The exclusion criteria were as follows: patients with (a) pseudoaneurysms involving the ascending aorta; (b) concomitant cardiac diseases that required open surgery; (c) anatomic features not suitable for TEVAR, such as severe stenosis of the access route arteries or a very large landing zone (> 40 mm) that limited device use; and (d) severe cardiopulmonary, renal, or hepatic diseases and thus could not tolerate general anaesthesia.

In total, 32 patients underwent chimney TEVAR according to the inclusion and exclusion criteria. The patients’ characteristics and comorbidities are listed in Table 1.

**Table 1:** Clinical demographics and characteristics of patients in this study

No.	Age (y)/Gender	BMI	Etiologies	Max diameter of aneurysm (mm)	Symptom	HTN	Hyperlipidemia	CHD	CRF	DM	Smoker	Other comorbidities
1	60/M	25.9	Trauma	64	None						√	
2	47/F	22.3	Trauma	48	Chest and back pain							
3	28/M	22.2	Trauma	42	None							
4	69/M	26.9	PAU or uncertain	43	None	√	√	√	√	√	√	Cerebral infarction, Disc protrusion, chronic bronchitis, emphysema
5	77/ M	26.1	PAU or uncertain	69	Chest and back pain	√	√	√	√		√	Hepatic insufficiency
6	65/M	28.6	PAU or uncertain	43	Chest and back pain	√	√					
7	74/M	28.6	PAU or uncertain	57	Hoarseness	√	√					Cerebral infarction, Disc protrusion; chronic bronchitis, emphysema
8	66/M	27.7	PAU or uncertain	57	Chest and back pain	√					√	
9	71/M	29.7	PAU or uncertain	30	None	√	√	√				Abdominal aortic aneurysm
10	29/M	21.7	Behcet’s disease	38	Chest and back pain							Behcet’s disease
11	67/M	25.2	PAU or uncertain	57	Chest and back pain	√	√	√			√	Gouty arthritis, chronic bronchitis; emphysema, lung nodule
12	76/M	24.5	PAU or uncertain	50	Hoarseness	√	√			√		
13	67/M	29.0	PAU or uncertain	53	None	√					√	
14	53/M	25.1	Trauma	32	None	√	√				√	
15	81/M	26.0	PAU or uncertain	40	Hoarseness	√		√		√	√	Left atrial enlargement
16	72/M	27.5	PAU or uncertain	61	None	√	√	√			√	Lung cancer, emphysema, gallstone, kidney stone,
17	53/M	26.1	PAU or uncertain	58	Hoarseness	√	√	√			√	
18	31/M	25.4	Trauma	55	Chest and back pain						√	
19	71/F	25.4	PAU or uncertain	30	Chest and back pain	√	√					Cerebral infarction, post-thrombotic Syndrome
20	81/M	26.5	PAU or uncertain	38	None	√		√			√	Atrial fibrillation
21	69/M	28.7	PAU or uncertain	61	None	√				√	√	carotid artery stenosis(< 70%)
22	74/M	26.2	PAU or uncertain	28	None	√		√				
23	71/M	25.0	PAU or uncertain	39	Hoarseness	√	√					
24	81/F	32.0	Trauma	52	None		√	√				Chronic bronchitis
25	66/M	24.4	Trauma	54	Hoarseness	√					√	
26	80/M	25.4	PAU or uncertain	56	None	√	√					Abdominal aortic aneurysm, hyperuricemia
27	46/M	28.4	PAU or uncertain	35	None	√	√					
28	50/M	19.0	Trauma	33	Chest and back pain						√	
29	60/M	27.7	PAU or uncertain	39	Chest and back pain	√					√	
30	76/M	23.5	Trauma	56	Chest and back pain		√			√	√	
31	58/M	22.8	Trauma	43	None						√	
32	80/M	26.4	PAU or uncertain	73	Hoarseness				√		√	

### Treatment procedure

For all 32 patients, blood pressure and heart rate were strictly controlled after admission (target blood pressure < 110/70 mmHg, heart rate < 70 beats/min). For the symptomatic patients, the systolic blood pressure was controlled to approximately 90/60 mm Hg, and the heart rate was 55–65 beats/min.

The chimney TEVAR procedure was performed in a hybrid operating room under fluoroscopic guidance. General anaesthesia with tracheal intubation was performed in all patients. The common femoral artery was exposed via surgical cut-down (26/29) and percutaneous puncture (3/29) using a Perclose ProGlide suture device (Abbott Laboratories Co., Ltd., USA), and if necessary, the brachial and carotid arteries were surgically exposed.

First, the chimney stent-graft was preloaded into the orifice of the target branch with the proximal side in the aortic lumen and the distal side maintained in the branch. Covered stents (Fluency; C.R. Bard, Inc., NJ, USA, or Viabahn; Gore & Associates, AZ, USA) were used as chimneys in all patients. Second, the aortic stent graft [Hercules (MicroPort Medical Co., Ltd., Shanghai, China); Zenith (Cook, Inc., Bloomington, IN, USA); Ankura (Lifetech Scientific Co., Ltd., Shenzhen, China); or Valiant (Medtronic, Inc., Minneapolis, MN, USA)] was inserted via femoral access and deployed in the preestablished position of the aortic arch. The relative position of the chimney and aortic stent-graft was adjusted to keep the chimney away from the lesion to avoid blood flow from the gutter to the pseudoaneurysm.

**Fig. 1:**
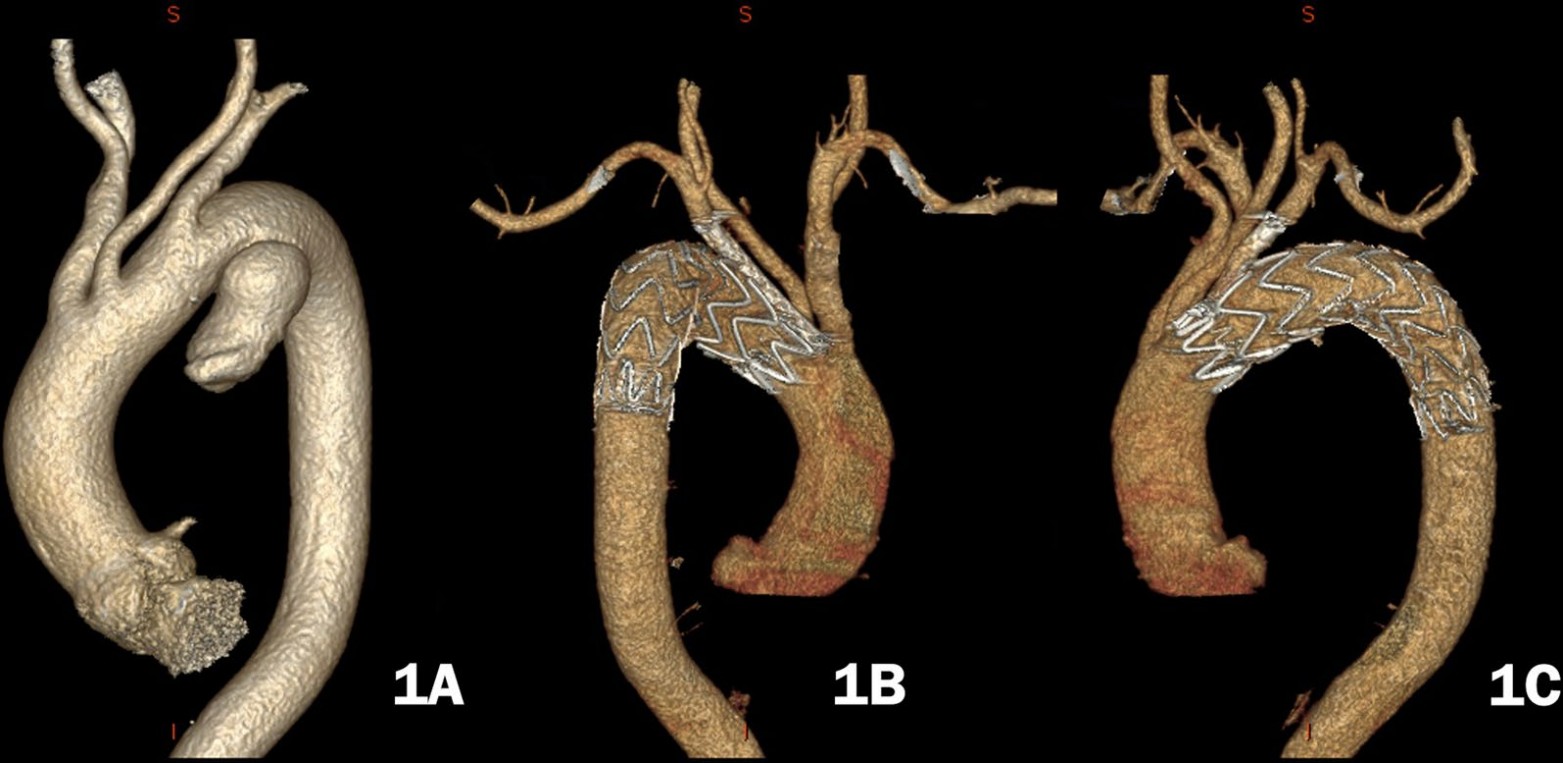
**A** a 55-year-old male had a pseudoaneurysm that involved the orifice of the left subclavian artery. **B** he underwent the single chimney technique for the left subclavian artery. **C** One year after the operation, the chimney stent graft remained patent, and the patient was in good condition

Third, the chimney was deployed with an approximately 10 mm proximal segment over the proximal fabric ending of the aortic stent-graft into the aortic lumen and the distal segment in the branch artery (Fig. 1). The chimneys in the innominate and carotid arteries were released immediately after deployment of the aortic stent graft to shorten the cerebral ischaemia time (often less than 1 min). For the purpose of improving long-term patency, chimneys were routinely dilated with a comparable balloon after deployment (10–12 atm, 5–10 s). After chimney TEVAR, digital subtraction angiography (DSA) was performed to confirm the final results. The aortic stent graft and chimney stent graft(s) were selected with 15–20% and 0–5% oversizing, respectively. Additional details of the operation process have already been reported previously by our colleagues [19, 20].

After chimney TEVAR, low-molecular-weight heparin (4,000 IU per 12 h) was administered for 3–7 days, followed by aspirin (100 mg/day) for at least 1 month. For patients without endoleaks, aspirin (100 mg/day) could be taken longer or even lifelong after chimney TEVAR, especially for those with a chimney less than 8 mm in diameter [4] or those with coexisting arteriosclerotic diseases that required antiplatelet therapy. For patients with type Ia endoleaks, aspirin was stopped until CTA showed satisfactory thrombosis of the gutter. The timing for the cessation of aspirin was decided comprehensively based on the order of severity of the endoleak, the chimney diameter and the coexisting diseases.

### Follow-up

All patients underwent CTA and duplex ultrasound scans 1 week after chimney TEVAR or before discharge to evaluate the exclusion results of pseudoaneurysm and supra-aortic branch patency. Subsequent follow-up with physical examination and CTA was scheduled at 3, 6, and 12 months and annually thereafter, and telephone follow-up was performed semiannually. The patients were asked to record all complaints and complications.

### Statistical analysis

All the data were enrolled and retrospectively analysed. Clinical data analysis was conducted with SPSS software (version 21, SPSS Inc., Chicago, IL), and GraphPad Prism (version 8; GraphPad Software, San Diego, CA, USA) was used to construct graphical representations of the data. The check of normality for continuous variables was performed, and the variables with normal distributions were expressed as the mean ± standard deviation, and those with a nonnormal distribution the median value was chosen. Kaplan‒Meier analysis was used to establish the rate of survival.

## Results

### Procedures

From October 2015 to August 2020, 32 patients with pseudoaneurysms underwent cTEVAR. The median age was 68.0 years (range, 28–81), and the aetiologies were as follows: trauma or a clear history of trauma in 10 (31.3%) patients, Bechet’s disease in 1 (3%) patient, and penetrating aortic ulcers or uncertain in 21 (65.6%) patients (Table 1).

Three (9%) patients underwent emergent surgery, which was defined as chimney TEVAR performed within 24 h after admission. The mean fluoroscopy time, which was defined as the period from the first to the last angiography, was 35.3 ± 7.7 min. The mean volume of contrast agent was 63.9 ± 7.0 mL. The mean length of intensive care unit (ICU) stay and postoperative stay were 2.2 ± 3.0 days and 8.3 ± 5.0 days, respectively (Table 2).

**Table 2:** Operative characteristics and outcomes

Characteristics	n (%)
Emergency cTEVAR	3 (9)
Aortic branches chimneys	
Left subclavian artery	29
Left common carotid artery	12
Innominate artery	3
Landing zones	
Zone 0	3 (9)
Zone 1	9 (28)
Zone 2	20 (63)
Fluoroscopy time (min)	35.3 ± 7.7
Contrast volume (ml)	63.9 ± 7.0
Post-OP ICU stay (days)	2.2 ± 3.0
Post-OP stay (days)	8.3 ± 5.0
Postoperative complications	
Endoleak	3 (9)
Spinal cord ischaemia	0
Retrograde dissection	0
Stroke/TIA	0
Chimney stenosis	1(3)
Death	4 (13)
Aortic related reoperation	0
Aortic related rehospitalization	0

TEVAR, thoracic endovascular aortic repair; OP, operation; ICU, intensive care unit; TIA, transient ischemic attack

**Fig. 2:**
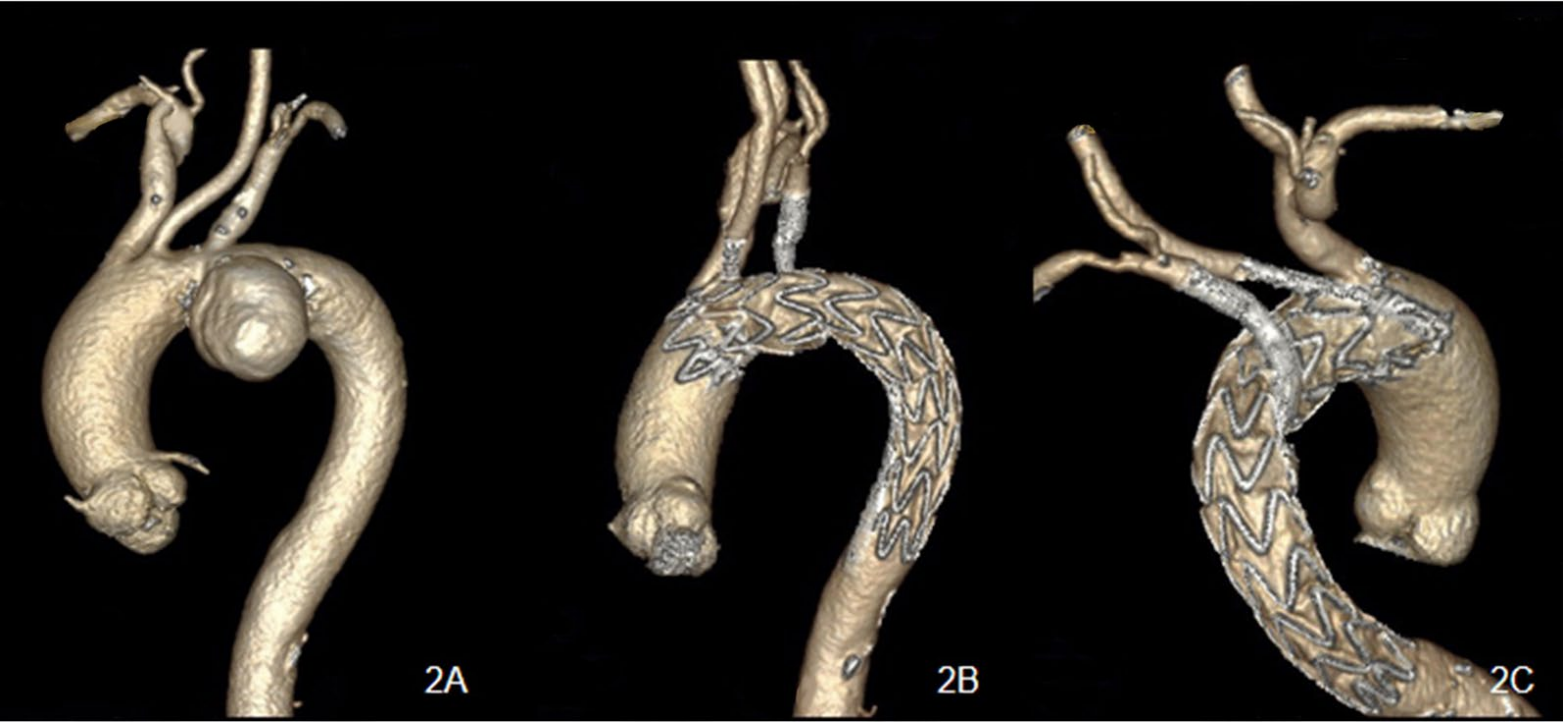
**A** a 75-year-old man with a huge pseudoaneurysm located at the aortic arch. **B** The patient underwent the periscope technique for the left subclavian artery and chimney technique for the left common carotid artery during thoracic endovascular aortic repair. **C** Two years after the operation, the chimneys remained patent, and the patient was in good condition

**Fig. 3:**
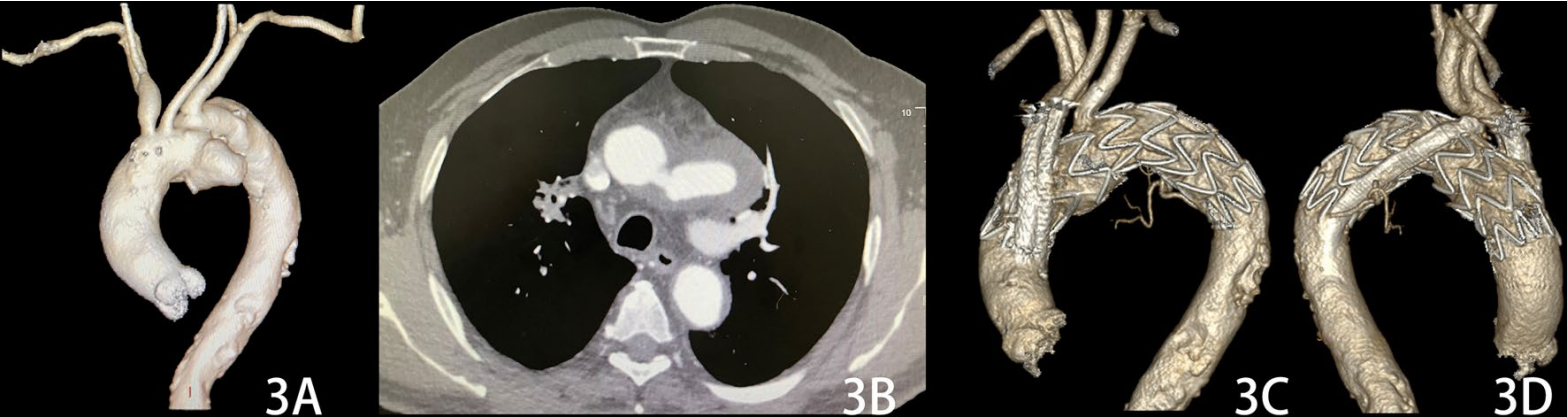
**A**, **B** The CT angiography of case 16 showed a pseudoaneurysm located at the aortic arch, and the patient underwent the triple chimney technique. **C**, **D**, CT angiography showed patent chimney stent grafts 18 months after the operation

In total, 48 chimney stent-grafts were implanted to preserve 44 target supra-arch branch vessels (Table 3). This included the LSA (n = 29), left common carotid artery (LCCA) (n = 12) and innominate artery (IA) (n = 3), and for 8 patients, the chimney technique for the LSA was performed in a reverse manner as a periscope (Figs. 2, 3). Moreover, in case 9, the patient simultaneously underwent endovascular repair of an abdominal aortic aneurysm with a maximum sac diameter of 60 mm, with special consideration that the patient had abdominal pain symptoms.

**Table 3:** Stent graft characteristics

Case	Aortic stent graft, mm	Landing zone	Chimney graft, mm		
			IA	LCCA	LSA
1	34-30-160 (LA)	2			8–60 (BF)
2	28-24-160 (LA)	2			8–60 (BF)
3	26-22-160 (MH)	2			10–60 (BF)
4	36-28-180 (LA)	2			8–60 (BF)
5	36-36-200 (MV)	0	14–60 (BF)	6–80 (BF)	8–150 (GV)
6	36-36-150 (MV)	2			6–60 (BF)
7	34-28-180 (LA)	2			8–60 (BF)
8	34-34-150 (MV)	2			8–80 (BF)
9	34-34-150 (MV)	2			8–60 (BF)
10	28-28-150 (MV)	2			5–50 (GV)
11	32-32-200 (MV)	1		8–60 (BF)	
12	32-32-200 (MV)	1		6–60 (BF)	
13	42-42-150 (MV)	0	10–60 (BF)	6–80 (BF)	
14	32-32-150 (MV)	2			8–60 (BF)
15	42-42-150 (MV)	2			6–60 (BF)
16	34-34-150 (MV)	0	8–60 (BF) 8–80 (BF)	6–80 (BF)	8–150 (GV)
17	32-32-150 (MV)	2			6–60 (BF)
18	28-22-180 (LA)	2			8–60 (BF)
19	34-30-160 (LA)	2			8–80 (BF)
20	34-30-160 (LA)	2			8–60 (BF)
21	36-32-160 (MH)	2			6–80 (BF)
22	34-34-150 (MV)	1		8–60 (BF) 8–80 (BF)	8–150 (GV)
23	34-30-160, 32-38-160 (MH)	1		6–60 (BF)	8–80 (BF)
24	42-42-200, 28-28-80 (MV)	2			8–50 (GV)
25	34-34-150 (MV)	1		6–80 (BF)	10–150 (BF)
26	34-24-200 (LA)	1		8–60 (BF)	8–50 (GV) 8–150 (GV)
27	30-24-160 (LA)	2			8–60 (BF)
28	28-28-150 (GT)	2			8–50 (GV)
29	32-32-150 (MV)	2			7–50 (GV)
30	30-30-200 (MV)	1		6–60 (BF)	6–150 (GV)
31	36-36-150 (MV)	1		8–60 (BF)	9–50 (GV) 8–150 (GV)
32	Valiant 30-30-150 (MV)	1		8–60 (BF)	6–150 (GV)

BF, Bard Fluency; GV, Gore Viabahn; LA, Lifetech Ankura; MH, MicroPort Hercules; MV, Medtronic Valiant; GT, Gore TAG; LSA, left subclavian artery; LCCA, left common carotid artery; IA, innominate artery

### Perioperative outcomes

Type Ia, Ib, II, III, and IV endoleaks occurred in 3 (9%), 0, 0, 0, and 0 patients, respectively. All type Ia endoleaks were slow flow and under close surveillance without reintervention. The detailed data related to the surgical procedure are shown in Table 2.

In our study, symptoms of hoarseness (n = 7) and chest and back pain (n = 12) were all relieved. All patients were given low-molecular-weight heparin and then a standard antiplatelet agent (100 mg/day aspirin) after chimney TEVAR, except for 3 patients who had type Ia endoleaks (case 1, case 10, and case 11). For them, antiplatelet therapy was cancelled 1 week (cases 1 & 10) and 1 month (case 11) after chimney TEVAR. After follow-up CTA showed no remaining endoleaks, antiplatelet therapy was given to the patients again thereafter. In case 10, the patient had Bechet’s disease and received standard immunotherapy postoperatively. For patients with hypertension, anti-hypertension medication therapy was continued perioperatively.

The 30-day mortality rate was 3% (n = 1). In case 5, the 77-year-old male patient had a large symptomatic aortic arch pseudoaneurysm with a maximum diameter of 78 mm that involved the whole arch. Because he was considered at very high risk for open chest surgery due to multiple severe coexisting diseases, TEVAR with the chimney technique for the IA and LCCA and a periscope for the LSA was performed. He had a history of exertional angina and was confirmed via CTA to have severe left main coronary artery stenosis, which was planned to be treated after endovascular aortic arch repair. Unfortunately, he died suddenly from acute myocardial infarction 10 days after the chimney TEVAR operation.

Another 67-year-old male patient, case 11, lived in the plateau area with an altitude over 3500 m for 30 years and suffered from a variety of diseases, including chronic obstructive pulmonary disease, pulmonary heart disease, pulmonary hypertension, multiple pulmonary nodules, hypertension, gout, and a history of bloody phlegm. The patient was transferred out of the ICU 2 days after the operation and transferred to the ICU again for rescue due to massive haemoptysis associated with bronchiectasis on the 7th postoperative day. After conservative treatment, he recovered and was discharged 26 days postoperatively. To date, he has lived uneventfully for an additional 5 years.

Patient 15 had a lower haemoglobin index (67 g/L) and left upper limb haematoma after the operation, and received brachial artery repair on the 15th postoperative day due to a pseudoaneurysm at the puncture point.

### Midterm follow-up

**Fig. 4:**
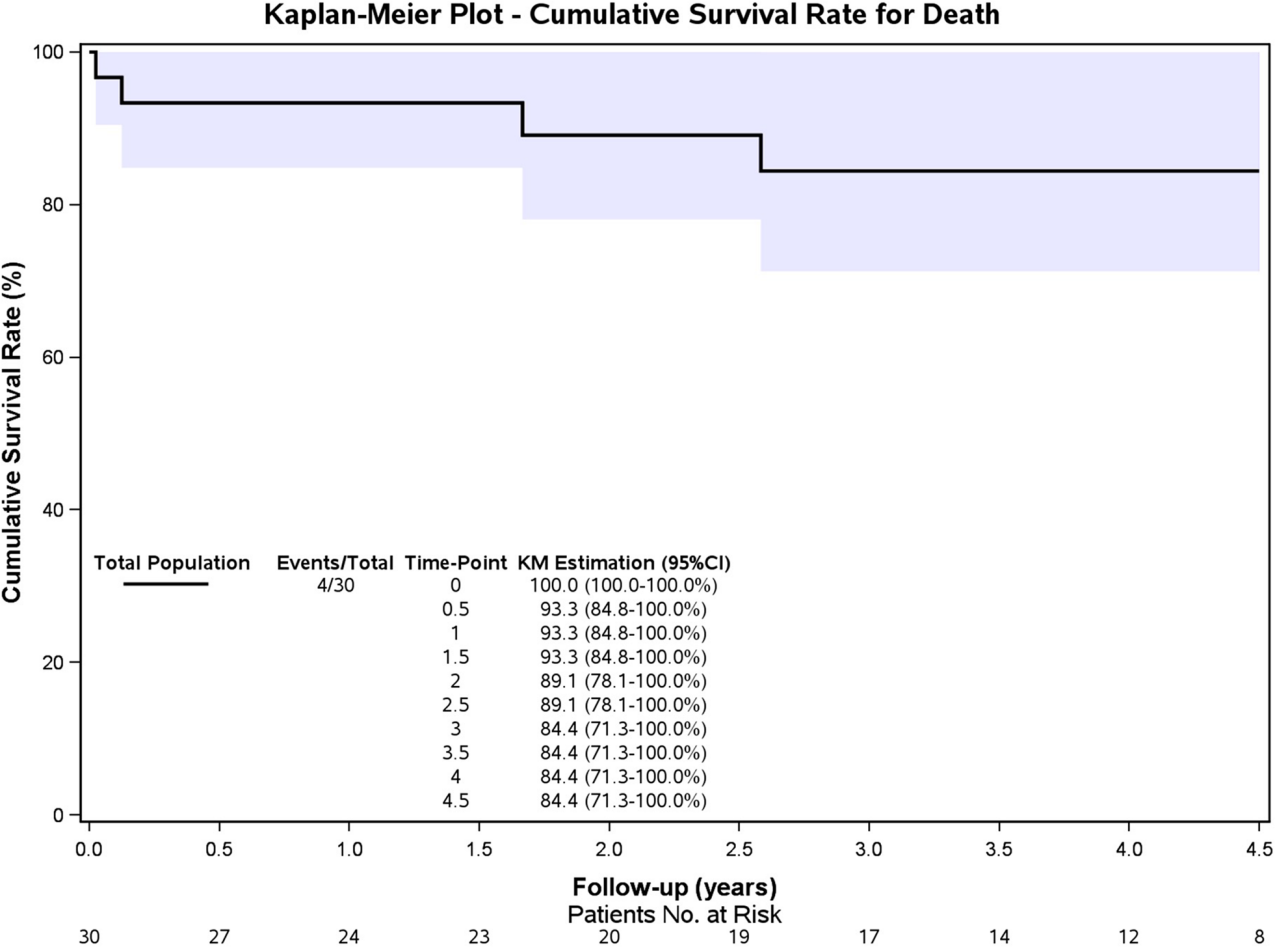
Kaplan‒Meier curve for overall patient survival. The 4.5-year survival estimate was 84.4% (95% confidence interval 71.3 to 100.0%)

Two patients were lost to follow-up, and the median follow-up was 46.5 ± 14.3 (range, 4.5–60) months. Three other patients died during the midterm follow-up period, and the Kaplan‒Meier survival curve is presented in Fig. 4. The overall 4.5-year survival rate was 84.4%. Patient 32 suffered from chronic renal insufficiency before admission, and he received regular dialysis treatment before discharge but died 1.5 months postoperatively. Patient 16 died in the 31st month after TEVAR because of lung cancer, and patient 24 died in the 20th postoperative month because of uncertain reasons.

**Fig. 5:**
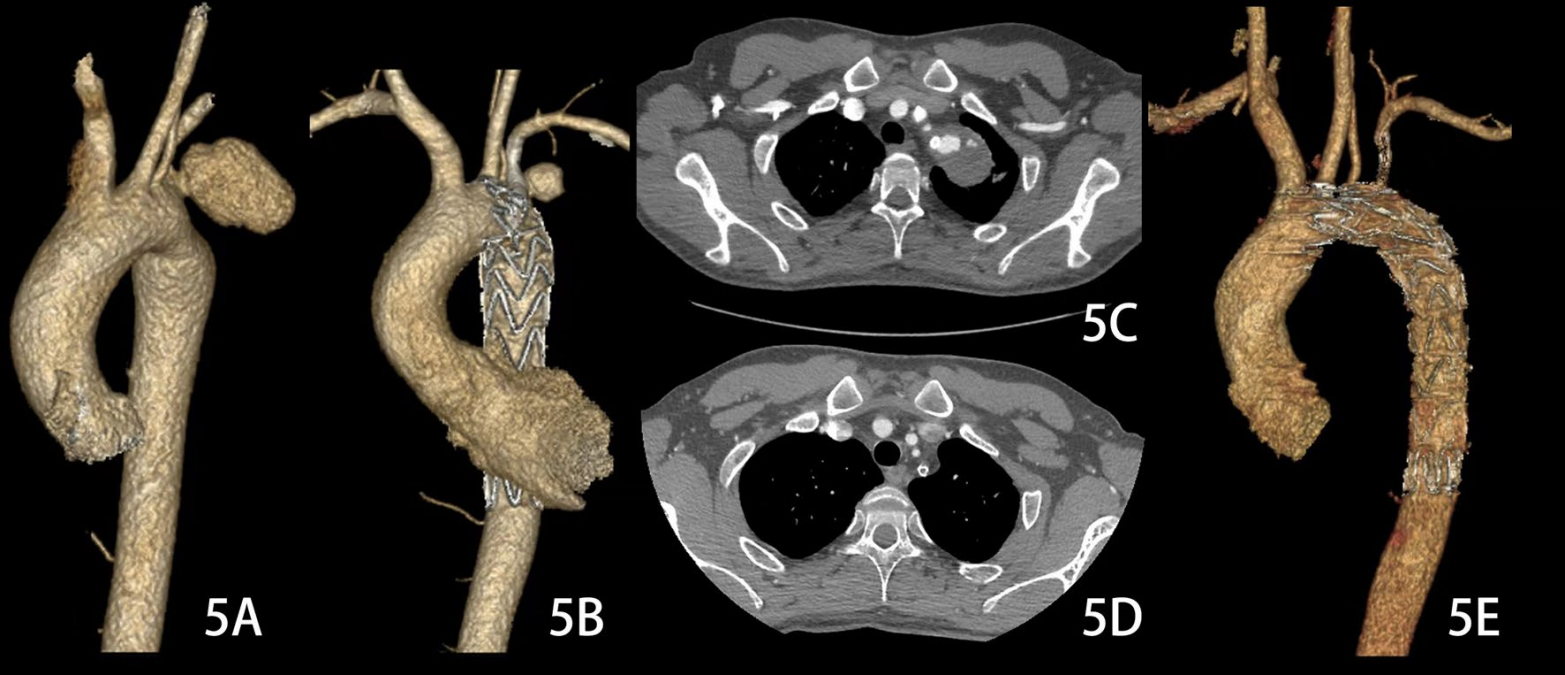
**A** The pseudoaneurysm was located at the aortic arch, which might be associated with Behcet's disease. **B**, **C** The patient underwent the chimney technique for the left subclavian artery during thoracic endovascular aortic repair, and a slow flow type Ia endoleak was observed 7 days after the operation. **D**, **E** One year after the operation, the endoleak disappeared, and obstruction of the chimney stent graft occurred. Because there was no obvious impact on the quality of life of his left upper limb, no further treatment was performed for the patient

With conservative treatment, including ceasing the use of antiplatelet agents and controlling the systolic pressure at 90–110 mm Hg and heart rate at 55–70 beats/min with antihypertensive agents and β-blockers, the 3 slow-flow type Ia endoleaks sealed spontaneously 3 months (case 1 & case 11) and 1 year (case 10, Fig. 5), respectively, after chimney TEVAR. However, the chimney stent graft of case 10 was occluded, which might have been associated with the cessation of antiplatelet agents, and 70% stenosis of the chimney occurred in case 18.

## Discussion

Aortic pseudoaneurysm is defined as a dilation of the aorta due to the disruption of all wall layers; it is only contained by periaortic connective tissue and can become a lethal situation, and the selection between endovascular and open surgical treatment depends on anatomic features, clinical presentations and comorbidities [1]. Some articles have reported the effectiveness of TEVAR in the treatment of pseudoaneurysms caused by tuberculosis [21, 22], trauma [3, 23], and Bechet’s disease [24]. TEVAR enabled minimization of the intraoperative risk, particularly in unstable multitrauma patients with a severe clinical status.

Traumatic aortic pseudoaneurysms occur in 2% of patients with blunt thoracic trauma [25, 26]. Endovascular treatment was used successfully early in 1997 by Semba [27]. A recent meta-analysis by Harky et al. [3] indicated that TEVAR carries lower in-hospital mortality and provides satisfactory perioperative outcomes compared with open repair in traumatic ruptured thoracic aortic pseudoaneurysms. Ten patients with possible traumatic reasons in this study had uneventful in-hospital and follow-up outcomes, and all had a history of traumatic accidents 5 to 20 years ago. The possible mechanism is that when the human body suffers deceleration injury, the arterial ligament may pull the wall of the small curvature of the aortic isthmus, which may lead to local injury, and over the years, the lesion progresses into a pseudoaneurysm as the blood flow impinges. Therefore, if the patient had unexplained pseudoaneurysms and a history of severe trauma several years ago (such as a car accident), the pseudoaneurysms might be related to the history of trauma.

For patients without aortic injuries, infections, or autoimmune diseases, PAU should be the most common aetiology for aortic pseudoaneurysms. The pseudoaneurysms might have originated from a calcified plaque rupture, and PAU could be the beginning form of pseudoaneurysms, which may lead to intramural haematoma, dissection, pseudoaneurysms, or even aortic rupture [28]. This pathologic condition is distinct from classic aortic dissection, which is more frequently seen in the natural history of PAUs with the propensity to evolve into aneurysms or pseudoaneurysms [29].

The chimney TEVAR technique was mostly used as a preferential choice in patients with inadequate proximal landing zones for standard TEVAR. The technique was used early by Criado et al. [30] in arch-TEVAR with bare stents to rescue LSAs in the landing zones. Hiramoto et al. [31] reported the administration of covered stents in the chimney technique assisted by TEVAR in 2006. According to our experience, covered chimney stents are very useful for decreasing the incidence of type Ia endoleaks.

The majority of the patients in our study received a single chimney graft. Compared with carotid-subclavian transposition or carotid-subclavian bypass, in chimney TEVAR, the incidence of neurological events is not high due to the shorter operation time and more minimally invasive neck incision. Therefore, we routinely performed chimney TEVAR instead of carotid-subclavian transposition or carotid-subclavian bypass. Our team reported the outcomes of the LCCA chimney technique for the endovascular repair of acute non-A–non-B aortic dissections in 2011 [4], and 8 patients were included in the study, with no mortality and a 100% chimney patency rate during a mean follow-up of 11.4 months. In 2015, a larger retrospective study of 41 patients reported by our team revealed similar perioperative results [19]. In 2017, Wang et al. reported the results of 122 patients (no pseudoaneurysms) who underwent chimney TEVAR [20], and the outcomes indicated that chimney TEVAR provided a safe, minimally invasive alternative with good chimney graft patency and low postoperative mortality for aortic arch pathologies. However, aortic arch pseudoaneurysms managed by chimney TEVAR have seldom been reported, and most previous studies in this area are case reports. This study significantly enriched the reported experience of chimney TEVAR for aortic arch pseudoaneurysms.

The risk of type Ia endoleaks is the main problem with the chimney technique because of the “gutter” between the chimney and aortic stent-grafts. We recommend an overlap of at least 2 cm between the chimney and aortic stent-grafts if possible. A longer overlap, adequate oversizing of the thoracic stent-graft and appropriate ballooning of the chimneys could narrow the gutter and decrease the incidence of type Ia endoleaks. If a pseudoaneurysm is restricted to one side of the aortic arch, such as the anterior or posterior wall, the relative position of the chimney and aortic stent graft should be adjusted to keep the chimney away from the lesion to avoid blood flow from the gutter to the pseudoaneurysm. When placing the chimney stent, the guide wire is introduced first for the establishment of the track. After the track is established, the relative position of the guide wire and the pseudoaneurysm will be observed by rotating the X-ray tube. If the wire and pseudoaneurysm are adjacent, there is a higher risk of endoleak. Therefore, a catheter with a curved head (such as a vertebral artery catheter) should be introduced. Then, part of the guide wire is retracted, and the guide wire preset at the distal position of the pseudoaneurysm with the help of a catheter. Finally, the chimney stent is introduced along the guide wire.

Aneurysms and pseudoaneurysms mainly differ in anatomy. In pseudoaneurysms, the majority of the aortic wall has been breached, but the extent of involvement is often focal, so pseudoaneurysms may have a lower incidence of type I endoleaks.

No cases of stroke occurred in our study. In our opinion, the most important factors for preventing stroke during and after chimney TEVAR include preoperatively analysing the target vessels with CTA and colour duplex ultrasound to see if there is any stenosis or calcified plaques, shortening the operation time via skilful manipulation, and open control instead of percutaneous puncture of the carotid or innominate artery, which may minimize vessel trauma and, accordingly, cerebral embolism risk.

Antiplatelet therapy including aspirin (100 mg/day) was administered routinely for patients with no endoleak after chimney TEVAR. However, for patients with endoleaks, especially type Ia endoleaks, cessation of antiplatelet therapy was considered individually. Antiplatelet therapy may be linked to an increased risk for the development of endoleak [32], and cessation of antiplatelet therapy may result in a risk of chimney occlusion and stroke. For patients with pseudoaneurysms, reducing sac pressure and avoiding rupture are the fundamental and key objectives of treatment. If the pseudoaneurysm pressure cannot be relieved and the safety of patients cannot be ensured, antiplatelet therapy must be cancelled to help thrombosis of the lesion. However, if severe type Ia endoleaks occur after the carotid or innominate chimney technique or if a patient has to use antiplatelet agents continuously for some reason (e.g., after coronary stenting), the decision of how to administer antiplatelet therapy would be very controversial. Therefore, it is important to emphasize that in patients with a high risk of endoleak, such as in cases where the target vessel arises from the pseudoaneurysm, the use of hybrid or other techniques instead of chimney TEVAR is recommended, especially for patients who cannot stop using antiplatelet agents.

## Limitations of this study

There are several limitations to this study. First, it was a retrospective and observational study, and the outcomes only represented the highly selected patients. Second, we classified these cases as pseudoaneurysms mainly based on imaging features and the medical history, such as trauma and Bechet’s disease, without pathological examination of the aortic aneurysm wall.

## Conclusion

Aortic pseudoaneurysm is a lethal pathologic condition. For highly selected aortic arch pseudoaneurysms with inadequate landing zones for TEVAR, the chimney technique seems to be feasible, with acceptable mid-term outcomes, and it could serve as an alternative minimally invasive approach to extend the landing zone.

According to this small cohort, slow flow type Ia endoleaks after chimney TEVAR could be conservatively treated. Additional experience is needed, and the long-term durability of chimney TEVAR requires further follow-up.

## Data Availability

The data used in this study are available from the corresponding author if needed.

## Abbreviations

TEVAR: Thoracic endovascular aortic repair
PAU: Penetrating aortic ulcer
CTA: Computed tomography angiography
LSA: Left subclavian artery
DSA: Digital subtraction angiography
ICU: Intensive care unit
LCCA: Left common carotid artery
IA: Innominate artery
CHD: Coronary heart disease
CRF: Chronic renal failure
DM: Diabetes mellitus
